# Unplanned Readmission prevention by Geriatric Emergency Network for Transitional care (URGENT): protocol of a prospective single centre quasi-experimental study

**DOI:** 10.1186/s12877-018-0933-x

**Published:** 2018-10-16

**Authors:** Els Devriendt, Pieter Heeren, Steffen Fieuws, Nathalie I. H. Wellens, Mieke Deschodt, Johan Flamaing, Marc Sabbe, Koen Milisen

**Affiliations:** 1 Department of Public Health and Primary Care, Academic Centre for Nursing and Midwifery, KU Leuven, Kapucijnenvoer 35/4, B-3000 Leuven, Belgium; 2 Department of Geriatric Medicine, University Hospitals Leuven, Herestraat 49, 3000 Leuven, Belgium; 3 Research Foundation Flanders, Egmontstraat 5, 1000 Brussels, Belgium; 4 I-Biostat Interuniversity Institute for Biostatistics and statistical Bioinformatics KU Leuven, Kapucijnenvoer 35/3, 3000 Leuven, Belgium; 5 Public Health and Social Affairs Department, Government Canton Vaud, Avenue des Casernes 2, 1014 Lausanne, Switzerland; 6 Department of Chronic Diseases, Metabolism and Ageing, Gerontology and Geriatrics, KU Leuven, Herestraat 49, 3000 Leuven, Belgium; 7 Department of Public Health, Nursing Science, University of Basel, Bernoullistrasse 28, 4056 Basel, Switzerland; 8 Department of Emergency Medicine, University Hospitals Leuven, Herestraat 49, 3000 Leuven, Belgium; 9 Department of Public Health and Primary Care, Emergency Medicine, KU Leuven, Kapucijnenvoer 35/4, 3000 Leuven, Belgium

**Keywords:** Acute care, Emergency department, Geriatric care model, Comprehensive geriatric assessment, Case management, Unplanned ED readmission

## Abstract

**Background:**

International guidelines recommend adapting the classic emergency department (ED) management model to the needs of older adults in order to ameliorate post-ED outcomes among this vulnerable group. To improve the care for older ED patients and especially prevent unplanned ED readmissions, the URGENT care model was developed.

**Methods:**

The URGENT care model is a nurse-led, comprehensive geriatric assessment based care model in the ED with geriatric follow-up after ED discharge. A prospective single centre quasi-experimental study (sequential design with two cohorts) is used to evaluate its effectiveness on unplanned ED readmission compared to usual ED care. Secondary outcome measures are hospitalization rate, ED length of stay, in-hospital length of stay, higher level of care, functional decline and mortality.

**Discussion:**

URGENT builds on previous research with adaptations tailored to the local context and addresses the needs of older patients in the ED with a special focus on transition of care. Although the selected approaches have been tested in other settings, evidence on this type of innovative care models in the ED setting is inconclusive.

**Trial registration:**

The study protocol is registered retrospectively with ISRCTN (ISRCTN91449949).

## Background

Twelve to 21% of all admissions to the emergency department (ED) are persons aged 75 years or above [1, 2]. Due to population aging, this percentage will substantially increase in the coming decades in a setting already burdened with crowding [3]. It has been demonstrated that managing older patients takes more time and resources, as they frequently suffer from multimorbidity, non-specific complaints and are at increased risk for adverse outcomes (e.g. nursing home admission, functional decline or death) [1, 2, 4]. Important factors contributing to these outcomes –which occur in one out of three discharged patients [5]- are presence of geriatric syndromes (e.g. delirium, functional impairment) and inappropriate discharge management [2, 6–8]. Yet, due to time constraints, architectural issues and lack of staff and expertise, limited attention is given to comprehensive evaluation of geriatric patients in the ED. Opportunities for improving care among these patients lie in changing the disease-oriented view towards a more comprehensive patient-oriented view [3, 9, 10].

Implementing comprehensive geriatric assessment (CGA) in ED care can improve the timely recognition of geriatric problems [8]. CGA has been defined as “a multidimensional interdisciplinary diagnostic process focused on determining a frail older person’s medical, psychosocial and functional capabilities in order to develop a coordinated and integrated plan for treatment and long-term follow-up” [11]. This approach has particularly shown a beneficial impact in patients admitted to acute geriatric wards [12–14]. The impact of CGA in ED patients remains inconclusive due to heterogeneity in interventions, study designs and ED settings [15–17].

Both American and European societies recently published guidelines for geriatric ED care [18, 19]. Besides structural, procedural and staffing recommendations, these guidelines also focus on transitional care between the ED and home care in order to prevent unplanned ED readmissions [18, 19]. These transitional care models combine the strengths of in-hospital CGA and structural collaboration with home care [20, 21]. The process from designing to evaluating such transitional care models is known to be complex and challenging; necessitating a step-by-step and structured approach. The aim of this paper is to describe the protocol of the ‘Unplanned Readmission prevention by Geriatric Emergency Network for Transitional care’ (URGENT) research project.

## Methods

### Study aim

The study aim is evaluating the effectiveness of the URGENT care model on unplanned ED readmission rate. Secondary outcomes are hospitalization rate, ED length of stay (LOS), in-hospital LOS, higher level of care, functional decline and mortality.

### Study design

**Fig. 1:**
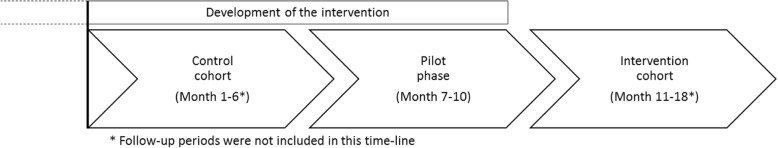
Timeline of the study

This study is designed as a prospective, single centre, quasi-experimental study (sequential design with two cohorts), in which usual ED care in the control cohort is compared to the URGENT care model in the intervention cohort (see Fig. 1).

### Setting

The study takes place in the ED of University Hospitals Leuven, one of the seven university hospitals in Belgium, counting 1995 beds. In 2016, 57,650 persons were admitted to the ED. Patients aged ≥70 years represented 28% (*n* = 15,892) of the total ED population. The admission rate of these group older patients is approximate 70%.

The ED of University Hospitals Leuven is organised as an unit with an admission section including triage, first aid, diagnosis and treatment (12 cubicles), and an observational unit of 30 beds including monitoring and intensive care beds. This observational unit aims at completing diagnostic tests and initial therapeutic interventions to determine the appropriate level of care within a period of time (generally 24 h) [22–24]. The ED has a half-open structure. Referred patients are seen by physicians belonging to the referred discipline. Non-referred patients are examined by one of the permanent available specialities: emergency medicine, general internal medicine, traumatology, paediatrics or psychiatry. A social care worker is also available during day hours. In Belgium, a referral by a general practitioner is recommended but not compulsory.

### Study population

Dutch-speaking, community-dwelling ED patients aged 70 years or older are eligible for enrolment. Patients are excluded if they live in a residential care setting, are transferred to the ED from an inpatient ward or another hospital, have a medical condition that makes an interview impossible, are unable to give informed (proxy) consent or are admitted to the ED on Saturday.

This study exclusively targets community-dwelling ED patients, because these patients might benefit the most from the intervention, since professional patient support is usual less intense at home compared to in a care facility (e.g. nursing home, rehabilitation clinic, hospital ward). In addition, patients to whom the geriatric intervention cannot be delivered within 36 hours after ED presentation are excluded, since longer door-to-intervention time is considered disadvantageous for possible intervention effects.

### Usual care

Patients in the control cohort receive usual ED care. A triage nurse screens the patient at the ED entrance and assigns the patient a priority level using the Emergency Severity Index (ESI) ranging from 1 (highest priority) to 5 (lowest priority) [25]. Subsequently, patients are seen in the admission section by a physician and a nurse for medical history taking, clinical examination and starting essential diagnostic testing and supportive or causal therapy. If there is a need for further observation or diagnostic work-up, a patient is transferred to the observational unit of the ED.

### Intervention

#### Development and pilot phase of the intervention

A multimethod approach, based on the MRC framework for developing and evaluating complex interventions [26], was used to design the URGENT care model. First, a literature review was conducted to identify key processes and structure outcomes associated with effective CGA-based geriatric interventions in the ED [27]. Second, a cross-sectional survey was conducted in all Belgian hospitals with an ED, focussing on care aspects, collaboration, education and infrastructure for older patients in the ED [28]. Third, observations, in-depth interviews and focus group meetings were conducted to map the experiences and expectations of patients, family members and caregivers towards care for older patients at the ED. Fourth, two prospective observational studies were conducted to compare characteristics of older patients admitted and discharged from the ED and to determine independent predictors for ED readmission [29–31]. The data of this study were used for several aspects of the URGENT project, such as sample size calculation and determination of the risk stratification strategy and the CGA content. Several stakeholders (i.e ED physicians, geriatricians, head nurses, ED nurses, geriatric nurses, managers, social workers, case managers) participated in focus group meetings to discuss how the international guidelines [18, 19] and the evidence of the described multimethod approach could be modelled into the URGENT care model. To achieve this, the project coordinators organized meetings with medical specialists (i.e. ED physicians and geriatricians), meetings with engaged paramedics (i.e. ED nurses, geriatric nurses, ED head nurse and social assistants) and meetings with the case managers and their supervisors.

A pilot phase of 4 months was conducted in order to look at acceptability and feasibility of the URGENT care model. The URGENT care model as described below was tested in this phase. Sixty-seven patients were piloted for the intervention. No fundamental changes were made to the protocol.

#### The URGENT care model intervention

The URGENT care model entails a nurse-led geriatric intervention, which is integrated in the standard ED care and comprises four additional consecutive steps: (1) identification of older patients at risk for adverse outcomes, (2) comprehensive geriatric evaluation, (3) interdisciplinary care planning and (4) geriatric follow-up. One full-time equivalent (FTE) dedicated geriatric emergency nurse (GEM nurse) is available from Monday to Friday between 9:00 AM and 5:30 PM to deliver the intervention on the ED. The background of the GEM nurses is either being an experienced nurse in the inpatient geriatric consultation team (0.5 FTE) or being an experienced ED nurse with additional geriatric training, provided by the inpatient geriatric consultation team (0.5 FTE). Although acceptability of the GEM nurse among ED staff was initially low in the pilot phase, this increased to a sufficient level through case-oriented collaboration.

##### Step 1: Identification of older patients at risk for adverse outcomes

Although there is no instrument that accurately predicts adverse events among older adults admitted to the ED [5], risk stratification is considered a necessary part of the intervention for two main reasons. First, not all eligible patients can benefit from the intervention. Second, resources are limited. Thus, to ensure that finite resources are allocated to at risk individuals, the interRAI ED screener is selected to make this discrimination. Its advantage compared to traditional screening tools is that its algorithm categorises patients in predefined strata. All patients within the high risk strata (i.e. screener score 5 and 6) automatically receive step 2 and 3, and if necessary step 4 of the intervention. Characteristics of these patients are having a functional impairment and a complex psychosocial context. Patients with a screener score 1 to 4 are considered as low risk patients. They will not receive other parts of the intervention, unless a member of the ED staff (i.e. nurse, physician or social care worker) argues that, based on his or her clinical judgement, the patient might benefit from CGA (i.e step 2). So, to identify older patients at risk for adverse outcomes, the URGENT project uses a two-track approach: the interRAI ED screener and clinical judgement.

The dedicated GEM nurse scores the interRAI ED screener and discusses clinical judgement as soon as possible after ED admission. Screening results are registered in the electronic medical record and are visible for ED staff in the electronic patient list.

##### Step 2: Comprehensive geriatric evaluation

The GEM nurse performs a standardised comprehensive geriatric evaluation including assessment of the functional, cognitive and social status of the at risk patient (see Table 1). The evaluation takes place after history taking and clinical examination, which is performed by a physician, or when the patient is waiting for further investigation or test results.

**Table 1:** Overview of comprehensive geriatric assessment in URGENT

Geriatric domain	Variables within the comprehensive geriatric assessment
Functional	❖ Activities of daily living (Katz index [35]) ❖ Fall History [34] ❖ Taking stairs ❖ Pain [34] ❖ Nutritional status: Appetite and weight loss [34] ❖ Alcohol use and smoking ❖ Medication intake ❖ Dyspnea [34]
Cognitive	❖ Screening for cognition: three-item word memory and clock drawing (Mini-Cog^©^ [36]) ❖ Orientation in time and place ❖ Attention ❖ Depressive symptoms (3-item screening tool for depression [38]) ❖ Screening for delirium (Confusion Assessment Method [37])
Social	❖ Age ❖ Gender ❖ Living situation (alone, together, other) [34] ❖ Formal care at home (e.g. nurse, meals on wheels, cleaning help, physiotherapist) ❖ Informal care at home (e.g. help from family, friends) ❖ Caregiver burden [34]
Medical	❖ Triage priority level (Emergency Severity Index [25]) ❖ Reason for admission ❖ First treating discipline on the ED ❖ Diagnosis ❖ Polypharmacy ❖ ED and hospital use in the last months

##### Step 3: Interdisciplinary care planning

Based on the results of the comprehensive geriatric evaluation, the dedicated GEM nurse formulates personalised advices and referrals and discusses these with the ED staff (nurse, physician, social care worker), the patient and his/her informal caregiver when available. These advices and referrals are based on setting specific protocols (e.g. criteria for admission, follow-up by the inpatient geriatric consultation team or referral to the geriatric day hospital) and can be divided into three categories: 1) advices and referrals to be followed during ED admission, 2) advices and referrals in case the patient is hospitalized and 3) post-discharge advices and referrals. An electronic report of this personalized interdisciplinary care plan is registered in the electronic patient record. In exceptional cases with high complexity, the dedicated GEM nurse can contact a geriatrician by phone.

##### Step 4: Geriatric follow-up

Follow-up during hospitalization or at home is provided if necessary. For patients admitted to a non-geriatric ward, an inpatient geriatric consultation team coordinates the in-hospital follow-up [32, 33]. Patients admitted to a geriatric ward receive geriatric in-hospital care according to the principles of an acute care for elders (ACE) unit [14]. For patients discharged home, an ambulatory consultation on the geriatric day clinic or case management at home can be planned. The aim of case manager follow-up is mainly providing clarification, assistance and coordination during the implementation of the personalized interdisciplinary care plan. The case manager is free of charge for the patient and collaborates with all formal and informal caregivers of the patient’s care team at home. Approval for post-discharge follow-up by the case manager is asked during ED admission or by phone within 7 days after ED discharge.

### Measures

#### Baseline variables

Demographic data on *gender* and *age* are collected by chart review. *Living situation* (living alone or together with) is asked during patient or proxy interview.

At ED admission, triage *priority level* and first treating *discipline* on ED *(surgical* versus *non-surgical)* are recorded. Triage priority level is measured by the *emergency severity index (ESI),* for patients admitted between 7 AM and 10 PM, meaning that patients admitted during night time have no recorded ESI score [25].

The *interRAI Emergency Department (ED) screener* is used to detect older patients at risk for adverse outcomes. This tool focusses on older patients’ functionality, self-rated health, self-rated mood, dyspnoea, unstable health conditions and caregiver burden [34]. The screener stratifies older patients into 6 risk levels in which level 1 represents low risk and level 6 high risk.

Functional status is measured using the *Katz Index of activities of daily living (ADL)* [35]*.* The scale consists of 6 items scoring the activities of daily living: washing, clothing, transferring, toileting, continence and feeding. Each item varies from 1 (independent) to 4 (dependent), which results in a total score ranging between 6 and 24. Functional status is registered at 4 different time points: prior to the event for which the patient visits the ED, at admission, 30 days after ED discharge and 90 days after ED discharge.

Following items of the interRAI ED Contact Assessment are retrospectively assessed during the index ED visit and scored dichotomously with yes or no: ‘Fall in the last 90 days prior to ED admission’, ‘daily and severe pain in the last three days prior to admission’, ‘weight loss of 5% or more in the last 30 days or 10% or more in the last 180 days’ and ‘caregiver burden’ [34].

Assistance for *instrumental activities of daily living (IADL)* before index ED visit (i.e. nursing care, home care, physiotherapy, meals on wheels, cleaning help, help for finances, help for medication intake and use of personal alarm system) is retrospectively assessed during the index ED visit.

*Polypharmacy* is defined as taking 5 or more drugs before the index ED visit. The polypharmacy score is based on the treating physician’s home medication registration during the index ED visit.

Screening for a cognitive deficit is done using *Mini-Cog*^©^. The Mini-Cog^©^ is a brief cognitive screening test and consists of a three-item recall task on three points and a clock drawing test on one point [36]. The test is positive (assumption of a cognitive deficit) if the score is two or less on four.

The *Confusion Assessment Method* (CAM) diagnostic algorithm is used to evaluate the presence of delirium. The CAM algorithm is based on the DSM-III-R criteria. Four characteristics of delirium are evaluated: 1) acute onset and fluctuating course, 2) inattention, 3) disorganized thinking, and 4) altered level of consciousness. A diagnosis of delirium according to the CAM requires the presence of characteristics 1, 2, and either 3 or 4 [37].

The 3-item screening tool for *depression* by Arroll et al. 2005 is used [38]. The first two questions are “during the past month have you often been bothered by feeling down, depressed or hopeless?” and “during the past month have you often been bothered by little interest or pleasure in doing things?” To reduce the number of false-positives, a third question was added that asks “is this something with which you would like help?” There are three possible responses to this question: “no”, “yes, but not today,” or “yes”. A patient is scored at risk for depression when reporting “yes” to at least two questions.

*ED visits or hospital admissions in the 90 days before the index ED visit* are registered during the patient interview and also retrospectively checked in the electronic patient files [34].

The ‘Modified Cumulative Illness Rating Scale’ (CIRS) is used to score *comorbidity*. This instrument consists of 14 biological systems [39]. Each system is scored from 0 to 4. A score 0 indicates ‘no problem affecting that system or past problem without clinical relevance’. Score 1 indicates ‘current mild problem or past significant problem’. Score 2 indicates ‘moderate disability or morbidity and/or requires first line therapy’. Score 3 indicates ‘severe problem and/or constant and significant disability and/or hard to control chronic problems (complex therapeutic regimen)’. Score 4 indicates ‘extremely severe problem and/or immediate treatment required and/or organ failure and/or severe functional impairment’. A total score (sum of all biological systems, with a maximum of 56) and a comorbidity index (number of biological systems with a score of 3 or more, with a maximum of 14) can be calculated.

#### Outcome variables

##### Primary outcome

The primary outcome of the URGENT project is *unplanned ED readmission.* This outcome is measured in all patients at 30 and 90 days after hospital discharge. Unplanned ED readmission is defined as a subsequent or repeat ED visit that followed the index ED visit or hospitalization, and could not have been foreseen at the time of discharge. *Time to ED return* is defined as the time from hospital discharge to an unplanned ED readmission. Follow-up time is restricted to 90 days.

##### Secondary outcomes

*Hospitalization rate* is defined as the relative number of patients who are hospitalized after ED presentation. This is measured in all patients.

Both *LOS* on the ED and in-hospital are measured. *ED LOS* is defined as the number of hours on the ED until discharge or hospitalization. *In-hospital LOS* is defined as the number of days in the hospital excluding the ED visit. To estimate the clinical impact of the intervention on LOS, automated hospital data of other patient groups (e.g. all adults, all individuals aged 70 years or more) can be used.

*Higher level of care* is defined as a professionally organized living arrangement that differs from the patient’s usual living place following ED or hospital stay (e.g. nursing home, a service flat, an advanced rehabilitation center, a psychiatric clinic, etc.). It comprises both temporary and permanent stays that are necessary since return to the usual living place is not achievable. This is measured for all patients at the moment of discharge. In the stratum ‘no hospitalization after ED visit’ higher level of care is measured at 30 and 90 days post-discharge, as well.

*Functional decline* is a decrease in physical functioning during follow-up. Physical functioning is measured by the Katz index (see baseline variables) and a decline is defined as an increase of 2 or more points on the total KATZ score during follow-up (at 30 and 90 days post-discharge) [40]. Mortality during follow-up will be considered as functional decline. This outcome is only measured in the stratum ‘no hospitalization after ED visit’.

*Mortality* is defined as the number of events (death) from the day of hospital discharge until 90 days post-discharge in all patients. Inhospital death was registered as well.

### Consent, enrolment and allocation

On weekdays, a study nurse recruits patients between 9:00 AM and 5:30 PM. Eligibility is checked by consultation of the electronic patient record (e.g. living situation) and by discussion with ED healthcare workers (e.g. medical condition). Patients are informed about the study and asked to participate. Proxies are informed about the study when appropriate and available (e.g. in case of cognitive problems). Written informed consent is obtained from all participants. Only among patients who are unable to write an explicit oral consent is accepted. Each participant (or proxy) receives written information.

### Data collection

Study nurses collect demographic and baseline data in the control cohort and among intervention cohort patients, identified as ‘low risk for adverse events’ (i.e. step 1 of the protocol). The dedicated GEM nurse collects these data among all at risk patients of the intervention cohort.

In both cohorts, study nurses register comorbidity scores and perform outcome registrations by review of the electronic patient file and by telephone calls at 30 and 90 days after ED discharge. The latter were only performed within the stratum ‘no hospitalization after ED visit’. Two study nurses independently score the comorbidity data. In case of disagreement, a medical expert is consulted to arbitrate.

Participants’ files and electronic data are stored securely at the study site (e.g. locked area, password protected hard- and software). Data integrity will be scrutinized with several strategies (e.g. valid values, range checks, consistency checks). Patient data are only identifiable with the unique participant’s hospital registration number. This variable will be deleted once the database is completed, making the dataset anonymous. All study protocol authors will have access to the anonymous dataset.

### Statistical methodology

#### Sample size calculation

Analysis of a recent cohort study’s data [29] reported an unplanned ED readmission rate of 27 and 23% within 12 weeks among the stratum of hospitalized and the stratum of non-hospitalized community-dwelling ED patients, respectively. A hospitalization rate of 70% and a 25% relative reduction of readmission rates (i.e. 20.25 and 17.25% readmission, respectively) were assumed, which approximately corresponds to a common odds ratio of 0.7. To detect this difference with at least 80% power, the required sample size based on a two-sided test (with α = 0.05) was 751 patients per cohort, making a total of 1502 patients. The calculation was performed with East software (East version 6.3). Sample sizes will be augmented with 10% to compensate for dropout. Note that the calculation is also an approximation since it is not based on a statistical approach, which handles potential differences in patient mix between both cohorts and the occurrence of deaths without readmission.

#### Propensity model

The potential difference in patient-mix, caused by the sequential design of the study, can induce bias in the comparison of both cohorts. To reduce the risk of bias, a propensity model is used, that weights each subject by its inverse probability of being in its specific cohort, conditional on a list of variables known during the ED stay and suspected a priori to be related to outcome. The objective is to create a weighted sample in which the distribution of the listed variables is the same between both cohorts. The probabilities of cohort membership are also known as propensity scores [41] and are obtained with a multivariable logistic regression model. Inverse probability of treatment weighting (IPTW) [42] is used to construct weights with the propensity score. The variables that are used in the multivariable logistic regression model to create the propensity scores are gender, age, ESI score, day of the week, time of presentation (hour), total CIRS score and first treating discipline on ED (surgical vs non-surgical).

#### Comparisons intervention with standard care

In all analyses (primary and secondary outcomes) the IPTW approach is used to handle the potential difference in patient mix between the cohorts.

##### Unplanned ED readmission

Relative risks are used for comparing the proportions of unplanned ED readmission at 30 and 90 days after hospital discharge. Since death without readmission is a competing risk for readmission, the cumulative incidence curve (using Nelson-Aalen estimates) is used to visualise the time until readmission instead of the Kaplan-Meier curve. Cox regressions stratified on hospitalized/not hospitalized are used to compare the time to unplanned ED readmission between both cohorts of patients being discharged alive from hospital. Cause-specific hazard-ratios are calculated based on the stratified analysis, as well as within each stratum separately. In this analysis, deaths without readmission are censored. Follow-up times (events and censored cases) are restricted to 90 days.

##### Other outcomes

Relative risks are used for comparing the proportions of following outcomes (at several time points when applicable): functional decline and higher level of care. Logistic regressions are used to compare hospitalization rate and mortality between both cohorts. A lognormal model is used to compare the ED LOS and the in-hospital LOS. Patients who die during hospital stay are excluded from the latter analysis. To handle the deaths within hospital stay, a Cox regression model is used to compare the cause-specific hazard (for hospital discharge) between groups.

### Time plan of the study

The control cohort is composed from month 1 to month 6. The intervention cohort is composed from month 11 to month 18. Between these two time periods, there is a gap of 4 months, in which the URGENT care model is piloted (Fig. 1). At the moment of protocol submission, data analyses are ongoing.

### Trial registration

The protocol for this study is registered retrospectively with ISRCTN (ISRCTN: 91449949). All items from the World Health Organization Trial Registration Data Set are available in Table 2.

**Table 2:** Overview of all items from the World Health Organization Trial Registration Data Set

Data category	Information
Primary registry and trial identifying number	ISRCTN registry ISRCTN: 91449949
Date of registration in primary registry	28 July, 2017
Secondary identifying numbers	B322201422910
Source(s) of monetary or material support	Flemish government agency for Innovation by Science and Technology (file number: 135182)
Primary sponsor	Flemish government agency for Innovation by Science and Technology (file number: 135182)
Secondary sponsor(s)	/
Contact for public queries	*Koen Milisen*, RN, PhD Academic Centre for Nursing and Midwifery, KU Leuven [koen.milisen@kuleuven.be] [0032 16 37 79 79]
Contact for scientific queries	*Koen Milisen*, RN, PhD Academic Centre for Nursing and Midwifery, KU Leuven [koen.milisen@kuleuven.be] [0032 16 37 79 79]
Public title	Unplanned Readmission prevention by Geriatric Emergency Network for Transitional care (URGENT)
Scientific title	Unplanned Readmission prevention by Geriatric Emergency Network for Transitional care (URGENT): protocol of a prospective single centre quasi-experimental study
Countries of recruitment	Belgium
Health condition(s) or problem(s) studied	Geriatric care needs
Intervention(s)	A step-by-step geriatric emergency care model is developed, piloted and implemented. Effectiveness of this intervention will be determined by comparing the intervention cohort with a usual care cohort.
Key inclusion and exclusion criteria	Dutch-speaking, community-dwelling ED patients aged 70 years or older are eligible for enrolment. Patients are excluded if they are transferred from a residential care setting, an inpatient ward or another hospital; have a medical condition that makes an interview impossible; are unable to give informed (proxy) consent or are admitted to the ED on Saturday. Patients to whom the intervention cannot be delivered within 36 hours after ED presentation are excluded, as well.
Study type	Interventional Quasi-experimental before-after study; sequential design with two cohorts (i.e. usual care cohort and intervention cohort) Primary purpose: prevention
Date of first enrolment	December 2014
Target sample size	1502
Recruitment status	Completed
Primary outcome(s)	Unplanned emergency department readmission
Key secondary outcomes	Hospitalization rate, ED length of stay, in-hospital length of stay, higher level of care, functional decline and mortality

## Discussion

This paper presents the methodology (i.e. study design, context, variables, development and description of the intervention, outcome measures, power calculation and analyses) of the URGENT study, that primarily aims to impact the unplanned ED readmission rate among older adults admitted to the ED.

The URGENT care model has been rigorously developed, guided by the MRC framework for complex interventions [26]. It integrates international guidelines [18, 19] and builds on previous research [27–30] with adaptations tailored to the local context. Stakeholder involvement (i.e. ED physicians, geriatricians, head nurses, ED nurses, geriatric nurses, managers, social workers, ICT experts, case managers) and pilot testing are used to increase acceptability and feasibility of the intervention before its implementation in the intervention cohort. Important findings of these partnerships are the choices to design a nurse-led GEM care model based on well-established concepts outside the ED setting such as CGA [14] and case management [43, 44]. Indeed, so far, the evidence for similar interventions in the ED setting has been non persuasive, warranting further research to develop and test this promising care models, such as the URGENT care model.

The research question to be answered requires a pragmatic approach. For example, the intervention is flexible due to the heterogeneity among the population of interest and because variations in adherence and preferences of patients and caregivers in the different settings (i.e the ED, inhospital care, home care) cannot be controlled. These contextual factors need to be part of the intervention to evaluate its implications for clinical practice properly in the end [45]. To achieve this, it is pivotal to work in a ‘real world’ setting and to see the study as a ‘system intervention’ at the level of the ED. Consequently, randomisation was not feasible, since this study is monocentric. Randomisation at patient level was waived as well due to the hypothesis that the GEM nurse will influence the knowledge and behaviour of ED staff –which would contaminate the control group. To reduce the risk of bias in the comparison of both cohorts, a propensity model is used. In combination with adequate sample size(s) and appropriate analysis techniques, the choice for a quasi-experimental design (sequential design with two cohorts) fulfils the methodological standards for evaluating the intervention’s effectiveness [45].

Dilution of the effect is an important risk within this study [45]. This can be caused by several aspects, such as the heterogeneity within the population and risk of inadequate compliance to the personalized interdisciplinary care plan. Besides the primary outcome, several care processes and secondary outcomes (i.e. hospitalization rate, ED LOS, in-hospital LOS, higher level of care, functional decline and mortality) are registered, as well. These might lead to generation of hypotheses, which can become subject of further research.

In conclusion, the URGENT care model is a rigorously developed and promising intervention with the potential to tackle the challenges among older patients in the ED and change the current management.

## Abbreviations

ADL: Activities of daily living
CAM: Confusion assessment method
CGA: Comprehensive geriatric assessment
CIRS: Modified cumulative illness rating scale
e.g.: example given
ED: Emergency Department
ESI: Emergency severity index
GEM nurse: Geriatric EMergency nurse
i.e.: id est
IADL: Instrumental Activities of Daily Living
IPTW: Inverse probability of treatment weighting
ISRCTN: International Standard Randomised Controlled Trials Number
URGENT: Unplanned Readmission prevention by Geriatric Emergency Network for Transitional care
